# A genome-wide screen reveals the involvement of enterobactin-mediated iron acquisition in *Escherichia coli* survival during copper stress

**DOI:** 10.1093/mtomcs/mfab052

**Published:** 2021-08-20

**Authors:** Kaitlin Casanova-Hampton, Alexis Carey, Sarah Kassam, Alyssa Garner, George L Donati, Shankar Thangamani, Sargurunathan Subashchandrabose

**Affiliations:** Department of Veterinary Pathobiology, College of Veterinary Medicine and Biomedical Sciences, Texas A&M University, College Station, TX, USA; Department of Veterinary Pathobiology, College of Veterinary Medicine and Biomedical Sciences, Texas A&M University, College Station, TX, USA; Department of Veterinary Pathobiology, College of Veterinary Medicine and Biomedical Sciences, Texas A&M University, College Station, TX, USA; Department of Veterinary Pathobiology, College of Veterinary Medicine and Biomedical Sciences, Texas A&M University, College Station, TX, USA; Department of Chemistry, Wake Forest University, Winston-Salem, NC, USA; Department of Pathology and Population Medicine, College of Veterinary Medicine, Midwestern University, Glendale, AZ, USA; Department of Veterinary Pathobiology, College of Veterinary Medicine and Biomedical Sciences, Texas A&M University, College Station, TX, USA

**Keywords:** Escherichia coli, copper, iron, enterobactin, uropathogenic *Escherichia coli*, TonB

## Abstract

Copper (Cu) is a key transition metal that is involved in many important biological processes in a cell. Cu is also utilized by the immune system to hamper pathogen growth during infection. However, genome-level knowledge on the mechanisms involved in adaptation to Cu stress is limited. Here, we report the results of a genome-wide reverse genetic screen for Cu-responsive phenotypes in *Escherichia coli*. Our screen has identified novel genes involved in adaptation to Cu stress in *E. coli*. We detected multiple genes involved in the biosynthesis and uptake of enterobactin, a siderophore utilized for high-affinity TonB-dependent acquisition of iron (Fe), as critical players in survival under Cu intoxication. We demonstrated the specificity of Cu-dependent killing by chelation of Cu and by genetic complementation of *tonB.* Notably, TonB is involved in protection from Cu in both laboratory and uropathogenic strains of *E. coli*. Cu stress leads to increased expression of the genes involved in Fe uptake, indicating that Fur regulon is derepressed during exposure to excess Cu. Trace element analyses revealed that Fe homeostasis is dysregulated during Cu stress. Taken together, our data supports a model in which lack of enterobactin-dependent Fe uptake leads to exacerbation of Cu toxicity, and elucidates the intricate connection between the homeostasis of Cu and Fe in a bacterial cell.

## Graphical Abstract

**Graphical Abstract:**
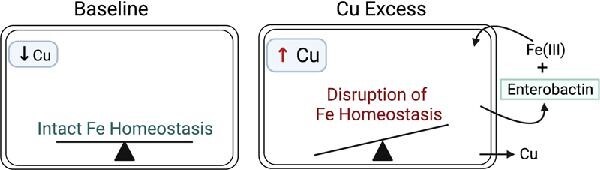
Copper Stress and Iron Homeostasis in *E. coli*.

## Introduction

Copper (Cu) is a transition metal that is utilized in various Cu-containing proteins in a bacterial cell. Cu homeostasis is a tightly regulated process, since excess Cu is toxic due to its potential to mismetallate proteins.^1,2^ Cu has higher affinity for noncognate ligands due to its location in the Irving–Williams series,^3^ and disrupts binding of transition metals such as iron (Fe), manganese (Mn), and zinc (Zn).^4^ Generation of reactive oxygen species used to be proposed as the mechanism by which Cu exerts its toxicity.^5^ However, recent studies have challenged this hypothesis by demonstrating that Cu can indeed protect *Escherichia coli* from peroxide and superoxide stress.^6,7^ Transcriptional regulators CueR and CusR play critical roles in orchestrating efflux and detoxification of excess Cu in *E. coli*.^8^ During Cu stress, Cu-bound CueR activates transcription of *copA* and *ceuO* encoding an inner membrane P-type ATPase, and a periplasmic multicopper oxidase, respectively.^9–11^ CusR, phosphorylated by CusS during Cu stress, induces transcription of *cusCFBA* operon that encodes the CusCBA transenvelope Cu efflux system and the CusF periplasmic cuprochaperone.^12^ Bioavailable Cu in the cytoplasm is maintained at subzeptomolar (<10^−^^21^M) levels by the CueR and CusR regulated genes, and sequestration of Cu by ligands.^8,13^ In addition, Cu stress activates other transcriptional regulatory systems including CpxRA and SoxRS, resulting in global changes that mitigate damage and promote survival of *E. coli*.^14,15^ While Cu efflux and detoxification systems in bacterial pathogens have been well characterized, uptake and trafficking of Cu in a bacterial cell remains less clearly defined.^13^

Antibacterial activity of Cu has been utilized by humans since the metal ages.^4,16^ Studies on multiple pathogens have revealed that bacterial Cu efflux/detoxification genes are involved in survival and virulence of those pathogens during infection.^16–18^ Cu is a key effector in the innate immune response against bacterial pathogens, with a clearly established antimicrobial function in the phagolysosomes of macrophages.^19,20^ Unabated increase in resistance to conventional antimicrobial agents in bacterial pathogens has led to a resurgence of interest in the development and use of Cu-based antimicrobial agents.^21^ Cu is increasingly used in health care settings on high-touch surfaces to mitigate microbial colonization and spread of nosocomial pathogens.^22–24^ It is critical to understand how a bacterial cell adapts to Cu stress to fully harness the antimicrobial potential of Cu, and to preemptively mitigate emergence of resistance. Transcriptional profiling studies have shed light on how gene expression is modulated during Cu stress in *E. coli*.^14,15^ However, the extent to which many of these Cu-responsive genes are involved in protection against cellular damage induced by Cu stress and their precise roles remains poorly understood.

To gain a comprehensive and genome-wide understanding of adaptation to Cu stress, we utilized the KEIO collection of genetically defined mutants lacking nonessential genes in *E. coli*^25^ to screen for Cu-responsive (sensitive or resistant) phenotypes. Our findings from this reverse genetic screen revealed that an intact enterobactin biosynthesis and uptake pathway is essential for optimal survival of *E. coli* during Cu stress. This study elucidates the central role of TonB in protection against Cu intoxication in both laboratory and pathogenic strains of *E. coli*.

## Methods

### Bacterial strains and culture conditions

Strains and plasmids used in this report are listed in Supplementary Table S1. Bacterial strains were cultured in LB broth or agar (tryptone 10 g/l, yeast extract 5 g/l, NaCl 5 g/l, and agar 15 g/l). Bacterial cultures were incubated at 37°C, and broth cultures were aerated by shaking at 200 RPM, unless noted otherwise. When indicated, spent medium was harvested from cultures in stationary phase by filter-sterilizing culture supernatants. Sterility of spent medium was assessed prior to use by plating on LB agar. Kanamycin (25 mg/ml) or ampicillin (100 mg/ml) was used for selection of mutants and ensure maintenance of plasmids. Chemicals were purchased from Sigma or Fisher Scientific.

### Primary screen for copper sensitivity and resistance

KEIO collection was used to start cultures in microtiter plates in LB. Optical densities at 600 nm were recorded in a plate reader (Omega Star, BMG lab tech). A 96-well pin replicator tool was used to stamp the overnight cultures onto LB agar with 0, 3, or 6 mM CuSO_4_ 5H_2_O. *Escherichia coli* strains BW25113 (parental strain for KEIO collection mutants), CFT073 (wild-type uropathogenic *E. coli*), CFT073 TN32-A2 (Cu-resistant mutant), and BW25113 *ΔcopA* (Cu-sensitive mutant) were also added on each plate as controls, and plates were incubated at 37°C and 24°C to detect the effect of temperature on Cu responsiveness. CFT073 TN32-A2 is a transposon mutant in uropathogenic *E. coli* strain CFT073 that has a Tn5 inserted in *c4289* encoding a predicted inner membrane protein (YhiM, Supplementary Table S1). This mutant exhibits a high level of Cu resistance compared to parental strain CFT073, and this phenotype could be reversed by genetic complementation (manuscript in preparation, Subash lab). Phenotypic response was determined qualitatively, relative to controls at 24, 48, and 72 h. Strains that exhibited a sensitive or resistant phenotype compared to the wild-type strain were tested in the secondary screen.

### Secondary and Fe Uptake/Metabolism Screen

During the secondary screen, overnight cultures were normalized based on optical density prior to inoculation on 3 and 6 mM CuSO_4_-containing LB agar plates at 37°C, 30°C, and 24°C. Phenotypic response was determined and documented at 24, 48, 72, and 96 h. Strains that exhibited a consistent sensitive or resistant growth compared to WT from three independent experiments were deemed as strains of interest. Thirty six mutants from the KEIO library containing a deletion in genes related to Fe uptake/metabolism (Supplementary Table S2) were screened in triplicate, essentially under the same conditions used in the secondary screen. All further assays for Cu-responsive phenotypes were conducted at 37°C with 24 h of incubation, as we did not detect temperature-dependent changes in our screens.

### CAS assay for catecholate siderophore production

Level of catecholate siderophore production was determined by CAS assays, as described recently.^26^ Briefly, overnight cultures of strains were spot plated on CAS agar plates, and zones of halo around the colonies were determined after 24 h.

### Validation of mutants from KEIO collection and expression of downstream genes in an operon

Oligonucleotide primers used in this study are listed in Supplementary Table S3. Primers flanking genes involved in Fe metabolism were designed and used in PCRs to confirm that the mutants utilized from the KEIO collection are indeed lacking the gene, and is replaced by the kanamycin cassette. KEIO mutants are designed not to disrupt expression of downstream genes, even when an upstream gene in an operon is replaced by antibiotic resistance cassette. Since many of the mutants of interest in Fe metabolic pathways are organized as operons, we conducted RT-PCR assays to verify whether the downstream genes were expressed. RNA was extracted from wild-type and mutant strains, reverse transcribed to cDNA, and used in PCRs to verify the presence of transcripts.

### Cu toxicity and chelation assays

Overnight cultures were normalized (OD_600_ = 1.0), diluted 1:100 in LB with or without 3 mM CuSO_4_, and cultured to stationary phase. When indicated, strains were cultured in spent media from stationary phase cultures of wild-type strain (BW25113). For chelating Cu, 6 mM bathocuproinedisulfonic acid disodium salt (BCS) was added. Viable counts were enumerated after 24 h.

### Genetic complementation of *tonB*

Full-length *tonB* and its native promoter were cloned into pUC57 (Genscript) and then subcloned into pGEN_MCS^27^ at BamHI and EcoRI sites to generate pGEN_*tonB* (Supplementary Table S1). Clones were verified by PCR with primers that bind to the vector (Supplementary Table S3). Empty vector and pGEN_*tonB* were introduced into the wild-type and *tonB* mutant strains by electroporation (Supplementary Table S1).

### Quantitative PCR

CuSO_4_ (0.5 mM) was added to cultures of wild-type strain in mid-logarithmic phase, and RNA was extracted after 20 min. Transcripts were stabilized with RNAprotect (Qiagen), extracted with RNeasy mini kit (Qiagen). Contaminating DNA was removed by digestion with DNase (Ambion), and cDNA was synthesized using SuperScript III reverse transcriptase (Invitrogen). Quantitative PCR was performed with SYBR Green (Thermo Scientific) and primers (Supplementary Table S3) in a CFX Real-Time system (Biorad Laboratories). *gapA* was used to normalize transcript levels between samples, and relative expression was calculated by using LB without Cu as the calibrator.

### Trace element analyses

Inductively coupled plasma-mass spectrometry or optical emission spectrometry (ICP-MS or OES) was used to determine transition metal content, as we have recently reported.^7^ Briefly, overnight cultures of bacterial strains were resuspended in LB with or without 3 mM CuSO_4_, incubated for an hour, harvested by centrifugation, and washed thrice with 10 mM HEPES containing 0.5 mM EDTA. Cell pellets were digested with trace element-grade nitric acid, and diluted in trace element-grade water. Levels of Cu, Fe, Zn, and Mn were determined by ICP-MS by an operator blinded to the treatment groups (8800 ICP-MS/MS, Agilent Technologies). Spectral interference was minimized by conducting the analysis in a single quadrupole mode using helium in the collision/reaction cell. Samples containing higher concentrations of Cu, Fe, and Zn were determined by inductively coupled plasma optical emission spectrometry (ICP-OES). The sample introduction system in the ICP-OES instrument (5110 SVDV ICP-OES, Agilent Technologies) was composed of a single-pass cyclonic spray chamber and a glass concentric nebulizer. Manufacturer default operating conditions were adopted in all ICP-OES analyses, and atomic/ionic emission signals at 327.395, 238.204, and 213.857 nm were used for Cu, Fe, and Zn determinations, respectively. Concentration of metals was normalized to the wet weight of *E. coli* pellets.

### Statistical analyses

Experiments were repeated at least three times independently, with two or more technical replicates. Results were analyzed in Prism 7 (Graphpad) with statistical tests indicated in *Figure Legends*.

## Results and discussion

### A genome-wide reverse genetic screen for Cu-responsive phenotypes in *E. coli*

**Fig. 1:**
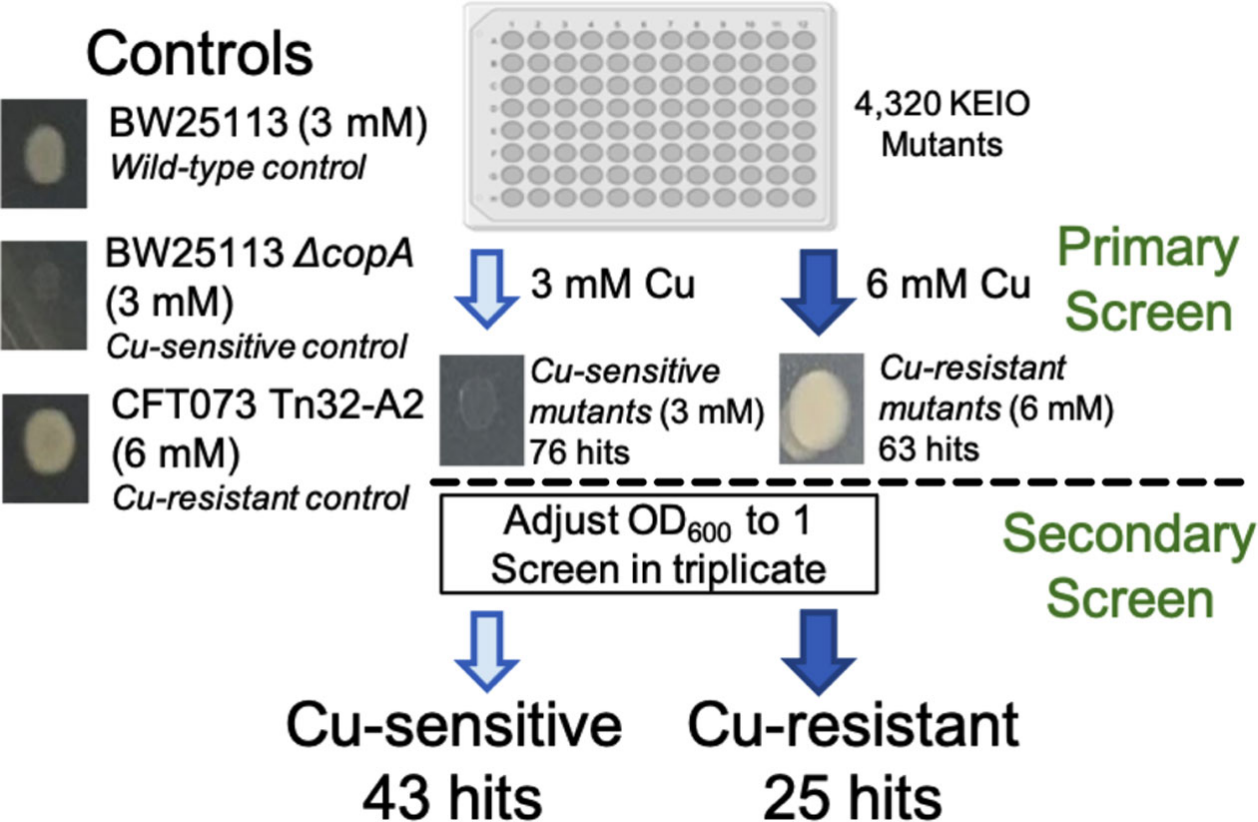
Detection of copper (Cu)-responsive *Escherichia coli* mutants. Schematic of the reverse genetic screen used to detect *E. coli* mutants from the KEIO library that are more sensitive or more resistant to Cu than the wild-type strain. Representative images of wild-type control strain BW25113 (3 mM Cu), Cu-sensitive *ΔcopA* mutant (3 mM Cu), and Cu-resistant CFT073 Tn32-A2 (6 mM Cu) mutants growing on Cu-supplemented LB agar from the primary screen are depicted here. Hits from the primary screen were validated in the secondary screen.

We utilized the KEIO collection of genetically defined mutants in *E. coli* strain BW25113, derived from K12 lineage, in this study.^25^ These mutants were generated by replacement of ORFs encoding nonessential genes with a kanamycin resistance cassette utilizing the lambda Red recombineering technology.^25,28^ Mutants (4320), and appropriate controls for wild-type, Cu-sensitive, and Cu-resistant phenotypes were screened at 3 and 6 mM CuSO_4_ in LB agar under aerobic conditions (Table 1 and Fig. 1). Based on our pilot assays with wild-type and control strains at various levels of Cu, we used 3 and 6 mM Cu in our screen for reproducible identification of sensitivity and resistance to Cu, respectively (Fig. 1). We utilized a rich medium in this screen to minimize the impact of conditional auxotrophy to select nutrients that develop during induction of Cu stress in minimal medium.^1,2^ Mutants that exhibited differential growth phenotypes, compared to controls, were verified in a secondary screen, which was conducted in triplicate. We identified a total of 43 and 25 mutants that were more sensitive and more resistant to Cu than the parental strain, respectively (Table 1, Supplementary Tables S4 and S5, and Fig. 1).

**Table 1.** Summary of screening for Cu-responsive phenotypes in *E. coli* KEIO mutant library

Screen	# of mutants screened	# of Cu-sensitive mutants	# of Cu-resistant mutants
Primary	4320	76 (1.8%)	63 (1.5%)
Secondary	139	43	25
Fe homeostasis	36	10	0

### Previously known and novel Cu-responsive genes detected in our screen

Our screen detected genes that were previously known to impart Cu-responsive phenotypes in *E. coli*, thereby validating the premise of this study and our findings (Supplementary Tables S4 & S5). Our results revealed that a Δ*lpp* mutant lacking the murein lipoprotein had higher Cu resistance (Supplementary Table S4), consistent with the findings of a previous report.^29^ We identified that a mutant lacking the Lon protease was more resistant to Cu than the wild-type strain (Supplementary Table S4). Our result supports the recent findings on CueR as a target of the Lon protease,^30^ as a Δ*lon* mutant would have constitutively high levels of CueR activity leading to a Cu-resistant phenotype. Mutants lacking an inner membrane Zn/divalent cation efflux ATPase ZntA—that have potentially higher intracellular accumulation of Zn—were also more resistant to Cu (Supplementary Table S4). Surprisingly, a mutant lacking the CusR transcriptional regulator that activates the transcription of *cusCFBA* genes involved in Cu efflux under anaerobic conditions was more resistant than the wild-type strain (Supplementary Table S4). This suggests that in the absence of CusR, another transcriptional activator likely induces the expression of the *cusCFBA* operon. Cross-regulation between CusRS and HprRS two component regulatory systems is known, and could contribute to the observed Cu-resistant phenotype.^15,31^ Multiple genes, including *ynaJ, yehS*, and *yciU*, whose function has not been characterized yet were also identified as contributors to Cu resistance in *E. coli* (Supplementary Table S4).

Our screen detected mutants lacking *copA* and *cueO* as Cu sensitive (Supplementary Table S5) in line with extensive data on the roles of these genes in Cu efflux and detoxification.^17^ Mutants lacking the transcriptional regulator CpxR also exhibited increased sensitivity to Cu (Supplementary Table S5) in line with previous reports.^32,33^ We observed that mutants lacking OmpR and OmpC (an OmpR-regulated porin) are more sensitive to Cu, suggesting that the potential for OmpC to act as an importer of Cu, as proposed in an earlier study,^34^ is less likely. Role of OmpR and OmpC in promoting resistance to Cu is aligned with recent observations on the role of these proteins in promoting Fe uptake in *E. coli*.^35^ We observed that several mutants lacking genes involved in Fe acquisition were more sensitive to Cu than the parental strain (Supplementary Table S5). A limitation of this screen is that regulators such as small RNAs that are often found in the intergenic regions will not be detected because they are not represented in the KEIO library. A forward genetic screen will be instrumental in identifying the role of regulators encoded outside of annotated genes in Cu-responsive phenotypes. Since our screen was conducted under aerobic conditions, genes involved in adaptation to Cu stress under anaerobiosis were not expected to be identified in this study. In summary, our screen has expanded the number of known Cu-responsive genes in *E. coli*, and represents a resource that lays the foundation for future studies on adaptation to Cu stress (Supplementary Tables S4 & S5).

### Mutants defective in enterobactin biosynthesis and ferric-enterobactin uptake are more sensitive to Cu

**Fig. 2:**
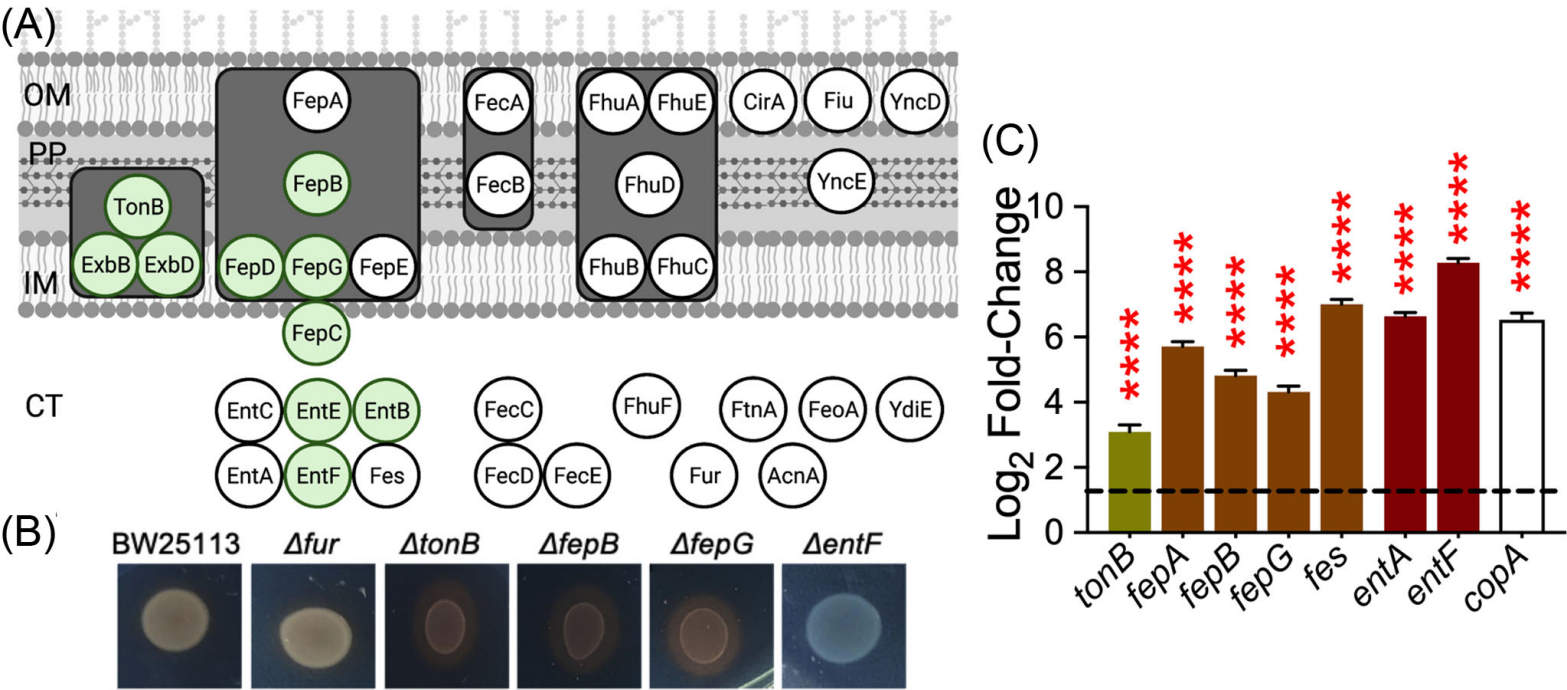
Mutants defective in enterobactin-dependent iron (Fe) uptake are more sensitive to copper (Cu), and transcription of Fe-uptake genes is induced during Cu stress. (A) Mutants lacking genes involved in Fe metabolism that was tested for sensitivity to Cu are depicted here, and clustered based on the pathway and cellular localization. Green circles indicate mutants that display increased sensitivity to Cu. OM, outer membrane; PP, periplasm; IM, inner membrane; CT, cytoplasm. (B) Production of enterobactin by select mutant and control strains was visualized on CAS agar. Presence of an orange halo/colony indicates biosynthesis and secretion of enterobactin. A representative image from three replicates is shown here. (C) Fold-change in the abundance of transcripts corresponding to enterobactin-mediated Fe-uptake system genes during Cu stress was determined. Cu-efflux gene *copA* was used as a positive control. All tested transcripts depicted above were *P* < 0.0001 by *t-*test in LB + Cu, compared to LB alone. N = 3, in triplicate. Mean + SEM is presented here.

Enterobactin is biosynthesized by products of the *ent* genes and is imported by the products of the *fep* genes in conjunction with the TonB-ExbB-ExbD complex.^17^ Six mutants with defects in uptake of ferric-enterobactin (Δ*tonB*, Δ*exbB*, Δ*exbD*, Δ*fepB*, Δ*fepD*, and Δ*fepG*) exhibited increased sensitivity to Cu in the secondary screen. We screened 36 mutants in the KEIO collection that are involved in enterobactin-mediated Fe acquisition pathway, and other known roles in Fe uptake/metabolism (Fig. 2A and Supplementary Table S2), for Cu-responsive phenotypes. Ten mutants belonging to two functional classes that are defective in enterobactin biosynthesis (Δ*entB*, Δ*entE*, and Δ*entF*), and ferric-enterobactin uptake (Δ*tonB*,  Δ*exbB*, Δ*exbD*, Δ*fepB*, Δ*fepC*, Δ*fepD*, and Δ*fepG*), were compromised in growth under Cu stress (Fig. 2A, Table 2, and Supplementary Table S5). Notably, none of these 36 mutants exhibited more resistance to Cu than the wild-type strain (Table 1 and Fig. 2A). We validated the deletion of specific genes in the mutants from the KEIO collection and assessed the expression of downstream genes in mutants lacking an upstream gene located within an operon by RT-PCR (Supplementary Table S6 and Supplementary Fig. S1). Next, enterobactin production phenotypes of these mutants were verified on CAS agar and compared to wild-type and Δ*fur* mutant strains (Fig. 2B). Fur is a repressor of transcription of genes involved in Fe uptake, including enterobactin biosynthesis and uptake.^36^ As expected, mutants defective in ferric-enterobactin uptake (Δ*tonB*, Δ*fepB*, and Δ*fepG*) produced more enterobactin relative to the parental strain, whereas the Δ*entF* mutant defective in enterobactin biosynthetic pathway did not produce enterobactin (Fig. 2B).

**Table 2.** Cu-sensitive mutants that are defective in enterobactin production and uptake

Gene	Function	Ent-production*^a^*	Ent-uptake^a^
*tonB*	TonB subunit complex, TonB	+	-
*exbB*	TonB subunit complex, ExbB	+	-
*exbD*	TonB subunit complex, ExbD	+	-
*fepB*	Ferric enterobactin ABC transporter periplasmic binding protein	+	-
*fepC*	Ferric enterobactin ABC transporter ATP binding subunit	+	-
*fepD*	Ferric enterobactin ABC transporter membrane subunit FepD	+	-
*fepG*	Ferric enterobactin ABC transporter membrane subunit FepG	+	-
*entB*	Enterobactin synthase component B	-	+
*entE*	2,3-dihydroxybenzoate-(aryl-carrier protein) ligase	-	+
*entF*	Apo-serine activating enzyme	-	+

^a^Ent, enterobactin; +, production/uptake competent; -, production/uptake deficient.

Our findings indicate that adaptation to high levels of Cu is associated with a disruption in cellular Fe homeostasis. Importantly, both enterobactin-deficient and overproducing mutants displayed increased sensitivity to Cu, pointing to divergent origins for the Cu-sensitivity phenotype. Nichols et al. have also detected ExbD as a contributor of optimal survival and growth of *E. coli* during Cu stress.^37^ However, loss of other Fe acquisition genes identified in our screen exhibited modest Cu-dependent decrease in growth in their assay. This difference could be a result of variation in the format of the screen with 1536 versus 96 colony format. Additionally, we conducted a screen with 36 mutants lacking various Fe uptake and metabolism genes that led to the confirmation and discovery of additional genes in the enterobactin-dependent Fe-uptake system in adaptation to Cu stress in *E. coli* (Fig. 2A, Table 2, and Supplementary Table S2). Grass et al. had reported that an *E. coli* mutant lacking Fur is conditionally hypersensitive to Cu in presence of ascorbate.^38^ Our assays were conducted in LB without added ascorbate, and is the likely reason that we did not observe Cu sensitivity phenotype in the *Δfur* mutant.

### Transcription of genes involved in enterobactin production and uptake are induced during Cu stress

Since mutants defective in production and utilization of enterobactin were more sensitive to Cu, we asked whether Cu stress induces transcription of the genes involved in enterobactin pathway. Transcription of these genes is repressed by Fur during growth in Fe-replete milieu and under anaerobic conditions, and derepression of transcription occurs upon starvation for Fe.^17,39^ Therefore, expression level of these genes could be used as an indicator of cellular bioavailability of Fe. Quantitative PCR was performed to assess the abundance of transcripts during Cu stress. Expression of *copA* was used as a positive control, since it is known to be induced during Cu stress (Fig. 2C). Genes involved in the biosynthesis (*entA* and *entF*) and uptake (*tonB, fepA, fepB, fepG*, and *fes*) of enterobactin were significantly upregulated in the wild-type strain during Cu stress, compared to growth in LB (Fig. 2C).

Our findings suggest that there is increased cellular demand for Fe during Cu stress leading to derepression of Fur regulon members. Alternatively, elevated Cu levels could interfere with transcriptional repression activity of Fur. As a Fe- and Zn-containing metalloprotein, Fur is a potential target for mismetallation by Cu. *Escherichia coli* Fur was recently demonstrated to reversibly bind [2Fe-2S] clusters that are less susceptible to mismetallation compared to [4Fe-4S] clusters.^40,41^ Our findings on the link between Cu stress and derepression of genes in the Fur regulon raise a critical question on whether Cu could directly disrupt Fe and/or Zn in Fur, and requires experimental verification. Recently, Zn excess has been demonstrated to increase cellular demand for Fe and a dysregulation of Cu homeostasis systems in *E. coli*.^42^ Taken in light of the results presented in this report, homeostasis of metals in microbes appears to be heavily interconnected, and has major implications for understanding microbial growth and physiology at the host–pathogen interface. Our results support a previous report on Cu-dependent transcriptional changes in *E. coli* that had identified upregulation of genes involved in Fe uptake, including the enterobactin pathway, upon exposure to Cu.^14^ Furthermore, Grass et al. have demonstrated elevated production of enterobactin during Cu stress in *E. coli.*^38^ We and others have reported that the expression of Fe-uptake genes is upregulated in uropathogenic *Escherichia coli* (UPEC) during clinical infection in people, which is also recapitulated in experimental animal models of UTI.^43–47^ Level of Cu is also elevated in urine during UTI caused by uropathogens.^43,48,49^ Similar to our findings in *E. coli*, enterobactin is also involved in optimal protection against Cu in *Salmonella enterica*.^50^ Taken in light of the observations presented in this report, further studies are required to dissect the contribution of Fe sequestration mechanisms from Cu intoxication within vertebrate hosts on derepression of Fur-regulated genes. Collectively, these findings establish that Cu intoxication is intricately linked with changes in Fe homeostasis in *E. coli*, a model organism and a pathogen of biomedical significance.

### Quantitative determination of Cu sensitivity

**Fig. 3:**
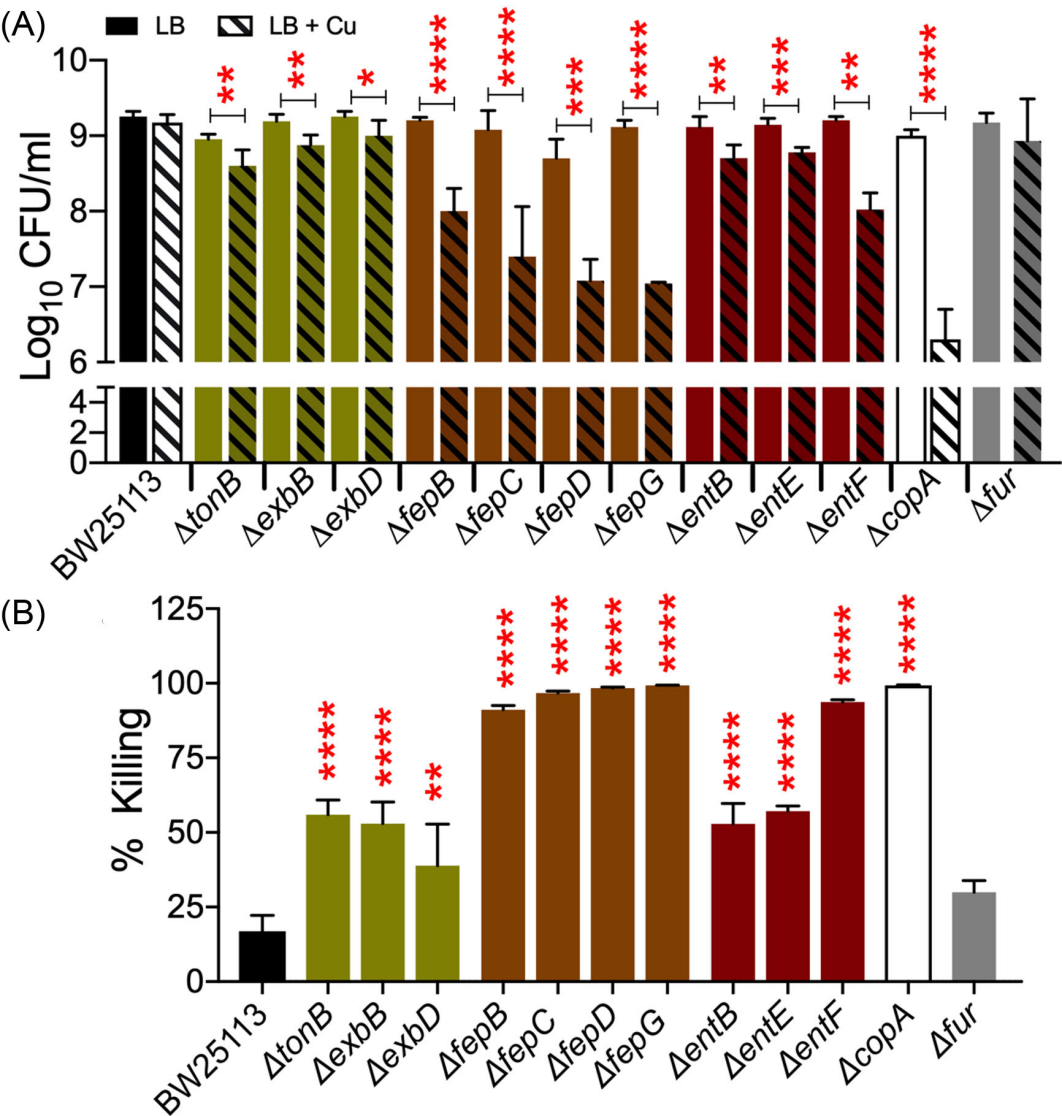
Viability of enterobactin-dependent iron-uptake mutants during copper (Cu) stress. (A) Wild-type (BW25113) and mutant strains were grown in LB without (solid bars) or with Cu (hatched bars), and viable counts were determined. Bars indicate median and error bars indicate interquartile range. (B) Percent killing was calculated from viable counts presented in panel A. Mean + SEM is presented here. Results from three independent experiments conducted in triplicate were analyzed by Mann–Whitney test (A), or ANOVA with Dunnett's test (B). ^*^P < 0.05, ^**^P < 0.01, ^***^P < 0.001, and ^****^P < 0.0001.

Since bacterial growth was evaluated qualitatively in our screens, we next determined the extent to which Cu decreases the viability of the mutants defective in enterobactin production and uptake. Wild-type and mutant strains were cultured overnight in LB with or without 3 mM Cu, prior to determining viable counts (Fig. 3A). A mutant lacking *copA* was used as a control to establish the presence of Cu stress in these assays (Fig. 3). A mutant lacking *fur* was used as a control for excess enterobactin production, compared to wild-type strain (Fig. 3). Quantitative plate counts revealed a significant decrease in viability for all tested mutants, except *Δfur*,  during Cu stress (Fig. 3A). There was 16% killing for the wild-type strain and 99% killing for the *ΔcopA* mutant in our assays (Fig. 3B). The mutant lacking *fur* exhibited wild-type level of viability during Cu stress (Fig. 3A and B). Killing of *ΔfepB, ΔfepC, ΔfepD, ΔfepG*, and Δ*entF* mutants by Cu was indistinguishable from that of the Δ*copA* mutant (Fig. 3B). Other mutants (Δ*tonB*, Δ*exbB*, Δ*exbD*, Δ*entB*, and Δ*entE*) were killed at a significantly higher level than the wild-type strain (Fig. 3B). While we expected increased Cu-dependent killing of Fe uptake-defective mutants based on our primary and secondary screens, we were surprised that some of these mutants exhibit *ΔcopA* level of sensitivity to Cu. The degree of augmentation in sensitivity to Cu in these mutants highlights the biological significance of enterobactin pathway in promoting *E. coli* survival during Cu intoxication. Our results also suggest that extracellular interaction of enterobactin with Cu does not mitigate Cu stress because enterobactin overproducing but import defective mutants including *ΔtonB,  ΔfepB*, and *ΔfepG* (Fig. 2B), exhibit higher level of killing by Cu (Fig. 3A and B) than the wild-type strain. The *Δfur* mutant, that overproduces and imports enterobactin, exhibits only wild-type level of killing (Fig. 3A and B) indicating that excess enterobactin does not augment protection from the toxic effects of Cu. Collectively, wild-type level of enterobactin production and import is sufficient to confer optimal protection against Cu stress.

### Specificity of Cu-induced killing in *E. coli*

**Fig. 4:**
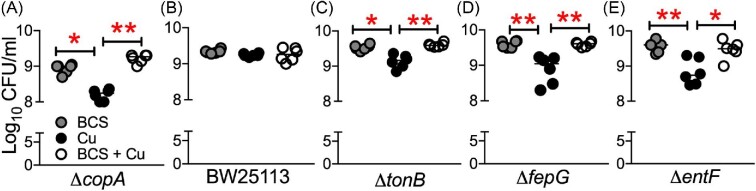
Chelation rescues growth of iron-uptake mutants during copper (Cu) stress. (A–E) Indicated mutant or wild-type strain was cultured in LB containing bathocuproine (BCS, 6 mM), Cu (3 mM), or BCS + Cu to stationary phase, and viable counts were determined. The entire experiment was conducted three times, independently, with each strain/condition tested in duplicate. Bars indicate median. ^*^P < 0.05 and ^**^P < 0.01 by Kruskal–Wallis test with Dunn's post-test.

We addressed whether increased killing during Cu stress is specific for Cu intoxication by using BCS, a known chelator of Cu^+^ ions. Our pilot assays indicated that BCS at up to 6 mM levels did not induce a detectible decrease in viable counts of wild-type, Δ*tonB*,  Δ*fepG*, Δ*entF*, and Δ*copA* mutant strains. Quantitative plate counts were performed from LB cultures incubated in the presence of BCS, Cu, or BCS + Cu (Fig. 4A–E). Supplementation of BCS rescued the growth defect of these mutants that was induced in the presence of Cu stress (Fig. 4A, C–E). Previously, yersiniabactin was demonstrated to confer additional protection from Cu toxicity in UPEC.^49^ Our findings indicate that the enterobactin system, encoded in the core genomes of *E. coli* and many other Gram-negative bacterial pathogens, plays a significant role in protection from Cu stress. Collectively, siderophores emerge as key players in cellular defense against the toxic effects of Cu in *E. coli*.

### TonB is required in uropathogenic *E. coli* to cope with Cu Stress

**Fig. 5:**
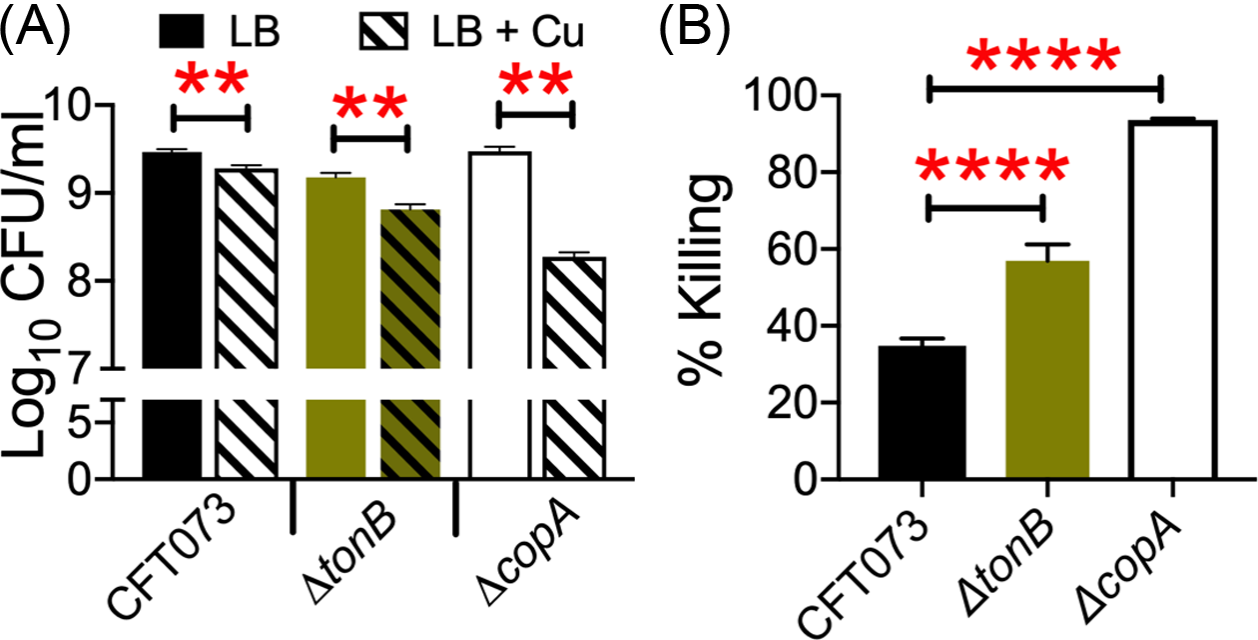
TonB is involved in protecting uropathogenic *Escherichia coli* from copper (Cu) stress. (A) Viable counts of wild-type (CFT073), *ΔtonB*, and *ΔcopA* mutant strains grown in LB without (solid bars) or with 3 mM Cu (hatched bars) is presented here. Bars indicate median and error bars indicate interquartile range. (B) Percent killing was calculated from panel A. Mean + SEM is presented here. Results from three independent experiments conducted in triplicate were analyzed by Mann–Whitney test (A) or ANOVA with Dunnett's test (B). ^**^P < 0.01 and ^****^P < 0.0001.

Laboratory strains of *E. coli* are reliant on enterobactin as the only siderophore-based Fe-uptake system.^51^ In contrast, pathogenic strains of *E. coli* produce both enterobactin and other siderophores including salmochelin, yersiniabactin, and/or aerobactin as redundant pathways to acquire Fe.^17,52,53^ We asked whether TonB, a central player in ferric-siderophore uptake, was also involved in protection from Cu toxicity in pathogenic *E. coli.* UPEC strain CFT073 was used because it produces salmochelin and aerobactin, in addition to enterobactin. A *ΔcopA* mutant in the CFT073 genetic background was used as a control in this assay (Fig. 5). Viability of wild-type strain and mutants lacking *tonB* and *copA* were tested in the presence and absence of Cu (Fig. 5A). All strains exhibited a statistically significant decrease in viable counts after growth under Cu stress (Fig. 5A). The *ΔtonB* mutant was more sensitive to Cu stress than the wild-type strain (Fig. 5B), reminiscent of our findings from the laboratory strain BW25113 (Fig. 3B).

TonB has been reported as a virulence factor in UPEC, since mutants lacking *tonB* and TonB-dependent Fe-uptake systems are attenuated in a mouse model of UTI.^53–55^ We have reported that endogenous host-derived Cu is involved in impeding UPEC growth in the murine urinary tract, since Cu-deficient mice are susceptible to higher bacterial burden during UTI than controls.^48^ Current observation on the role of TonB in protection from Cu intoxication suggests that Fe acquisition is not only critical for continued UPEC growth within the host, but also required for optimal survival under Cu stress at the host–pathogen interface. Small molecules that specifically inhibit the activity of TonB in UPEC have been described.^56^ It will be of interest to test whether these TonB inhibitors modulate the sensitivity of UPEC to Cu, and their impact on modulating UPEC colonization in an experimental model of UTI.

### Genetic complementation restores Cu resistance in the *ΔtonB* mutant

**Fig. 6:**
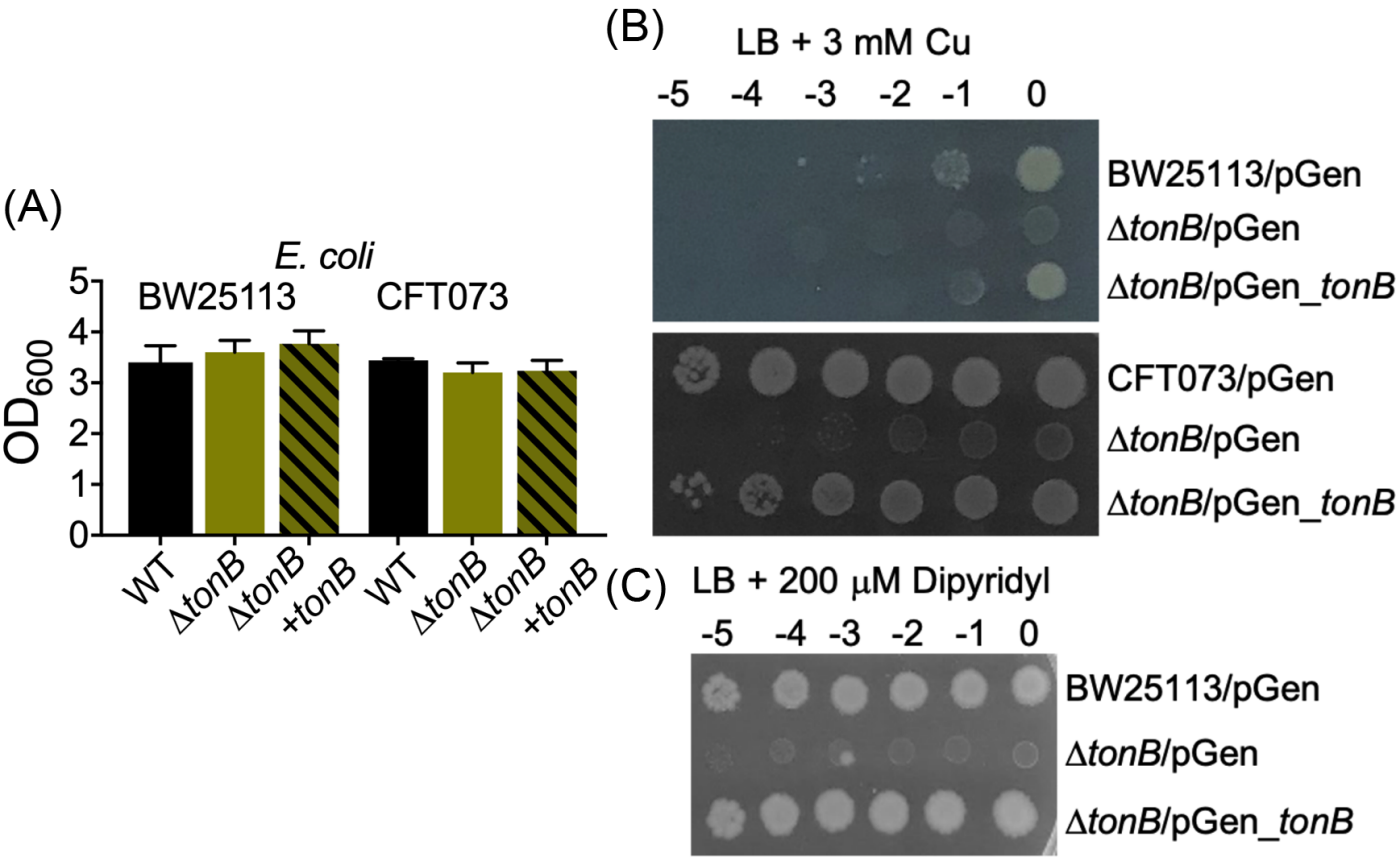
Genetic complementation of the *ΔtonB* mutant. (A) Wild-type (BW25113 or CFT073) and *ΔtonB* mutant with empty vector (pGen), and the complemented mutants (pGen_*tonB*) were cultured overnight in LB and optical density was determined at 600 nm. (B) Serial 10-fold dilutions of overnight cultures, from panel A, were plated on LB with 3 mM Cu and images were acquired after 24 h of incubation. The experiments were repeated thrice, and a representative image is depicted. (C) Complementation of *tonB* reinstates growth of the *ΔtonB* mutant in the presence of dipyridyl, a known iron chelator.

To establish a causal link between mutants defective in Fe uptake and increased Cu sensitivity, we took a genetic complementation approach. We selected *tonB* for genetic complementation since many other enterobactin pathway genes are organized as lengthy operons, which makes complementation under the transcriptional control of native promoters a challenging task (Supplementary Fig. S1). Full-length *tonB* and its native promoter was cloned into a low-copy number vector and introduced into the *ΔtonB* strains (Supplementary Table S1). To rule out strain-specific effects, we tested the role of *tonB* in Cu resistance in both laboratory (BW25113) and uropathogenic *E. coli* (UPEC CFT073) strains (Fig. 6). Wild-type, mutant, and complemented mutant strains had comparable turbidity (OD_600_) during growth in LB in the absence of Cu stress (Fig. 6A). Reintroduction of *tonB* in *trans* rescued growth of the *ΔtonB* mutant in the presence of Cu in both laboratory (BW25113), and pathogenic (CFT073) strains of *E. coli* (Fig. 6B). To further confirm the specificity of this phenotype, we tested the growth of these strains under Fe limitation. Genetic complementation reinstated the ability of *ΔtonB* mutant to grow in the presence of dipyridyl (Fig. 6C).

### Copper stress is mitigated in *E. coli* during growth in spent culture medium

We utilized culture supernatants in lieu of chemical complementation to further investigate the role of enterobactin biosynthesis and uptake in adaptation to Cu stress. The *ΔtonB* and *ΔentF* strains exhibited significantly lower growth in spent medium harvested from stationary phase cultures of wild-type strain compared to the wild-type strain (Supplementary Fig. S2). However, the *ΔtonB* strain had significantly lower growth in spent medium supplemented with Cu compared to wild-type and *ΔentF* strains (Supplementary Fig. S2). Interestingly, the WT and *ΔentF* mutant strains, that are competent for importing enterobactin, exhibited comparable growth in spent medium supplemented with Cu. These findings on the rescue of growth of an enterobactin biosynthesis defective mutant (*ΔentF*), but not an enterobactin import defective mutant (*ΔtonB*), in the presence of Cu in spent medium suggests a role for enterobactin import in adaptation to Cu stress. However, this experiment has to be evaluated with pure enterobactin to assess the specificity of our findings.

### Cellular transition metal content during Cu stress

**Fig. 7:**
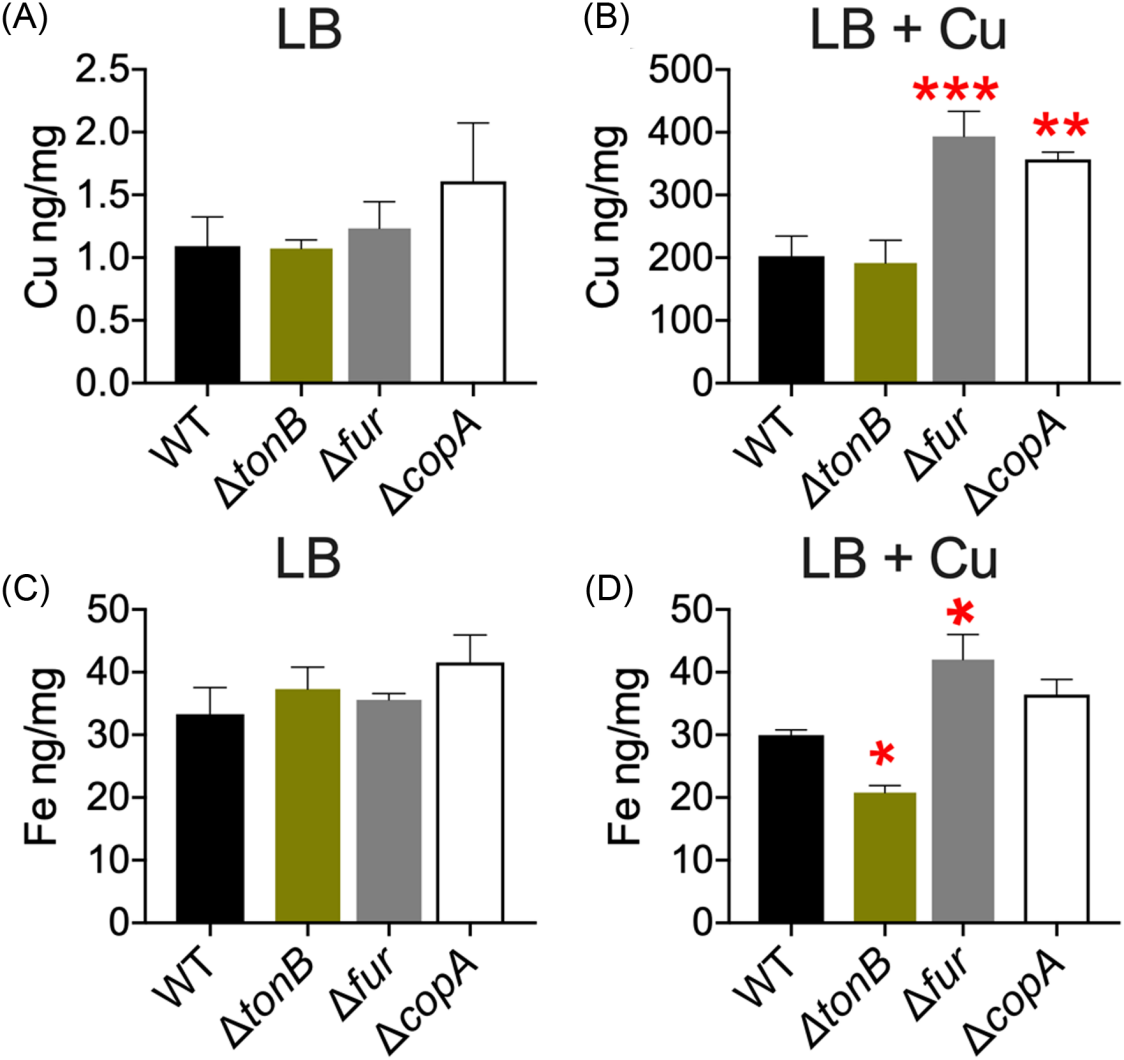
Cellular transition metal content during copper (Cu) stress. Wild-type (BW25113), *ΔtonB, Δfur*, and *ΔcopA* mutant strains were exposed to 0 or 3 mM Cu in LB. Cell pellets were digested and analyzed by inductively coupled plasma-mass spectrometry or optical emission spectrometry (ICP-MS or OES) to quantify Cu (A and B), iron (Fe) (C and D). Mean + SEM of data from three biological replicates is presented here, and analyzed by ANOVA with Dunnett's test. ^*^P < 0.05, ^**^P < 0.01, and ^***^P < 0.001

To test whether these mutants are compromised in their ability to maintain normal level of transition metals, we determined the cellular content of key transition metals (Cu, Fe, Zn, and Mn) by ICP-MS or OES. There was no detectible difference in the content of these metals in the wild-type, *ΔtonB, Δfur*, and *ΔcopA* strains grown in LB (Figs. 7A and C, and Supplementary Fig. S3A and C). Mutants (*Δfur* and *ΔcopA*) were used as controls for dysregulation of Fe homeostasis and loss of cytoplasmic Cu efflux, respectively (Fig. 7 and Supplementary Fig. S3). As expected, Cu content was increased in the wild-type and mutant strains grown in Cu-supplemented medium, compared to LB (Figs. 7A and B). Cellular Cu content in the *ΔtonB* mutant was indistinguishable from that of the wild-type strain in LB with and without additional Cu (Fig. 7A and B). We observed hyperaccumulation of Cu in the *Δfur* mutant comparable to that of a known Cu-hyperaccumulating *ΔcopA* mutant strain, and significantly higher than the wild-type strain (Fig. 7B). Cellular levels of Fe were similar in these strains in the absence of Cu stress (Fig. 7C). We noted a significant decrease in cellular Fe load in the *ΔtonB* mutant relative to the wild-type strain (Fig. 7D). On the contrary, and as expected, Fe content was elevated in the *Δfur* mutant (Fig. 7D). Although the *ΔcopA* mutant had higher level of Fe than the wild-type strain, this difference was not statistically significant (Fig. 7D). The *ΔcopA* mutant had accumulated significantly higher level of Zn than the parental strain during Cu stress (Supplementary Fig. S3B). There were no significant changes in cellular Mn load in these strains during Cu stress (Supplementary Figs. S3C and D).

**Fig. 8:**
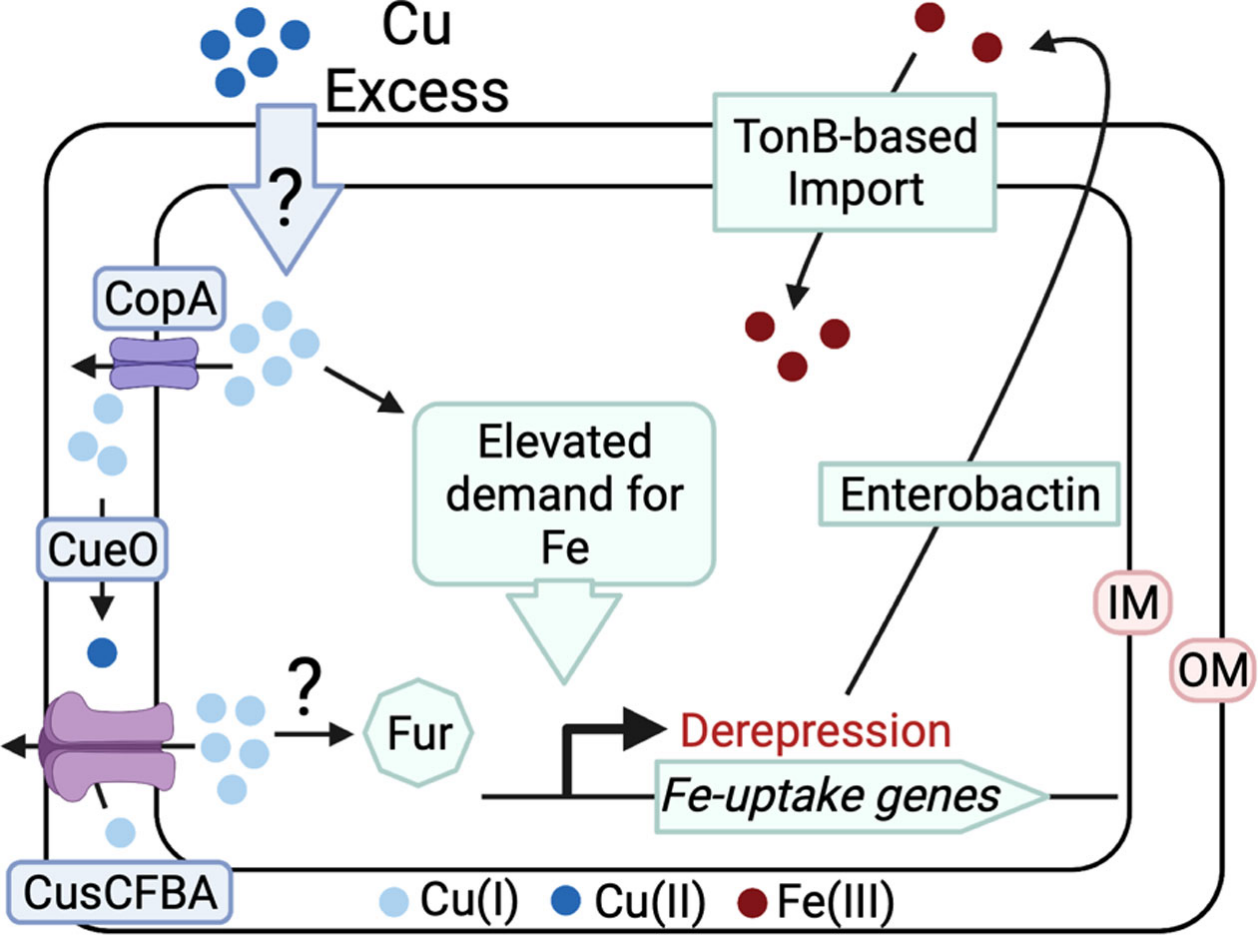
Role of enterobactin in protecting *Escherichia coli* from copper (Cu) stress. Intracellular Cu levels increase during Cu stress leading to increased demand for iron (Fe) associated with disruption of Fe homeostasis. Accumulation of Cu in the cytosol triggers expression of Cu efflux and detoxification systems encoded by the *copA, cueO*, and *cusCFBA* genes. Elevated cellular need for Fe results in derepression of Fur-regulated genes that culminates in the production of enterobactin and uptake of ferric-enterobactin. The ability to ramp up Fe import protects wild-type *E. coli* from Cu intoxication, and mutants defective in production/uptake of enterobactin are compromised in survival during Cu stress. IM, inner membrane; OM, outer membrane.

Our ICP-MS and OES analyses suggest that the enhanced sensitivity of the *ΔtonB* mutant is likely due to lower Fe content relative to the wild-type strain, although both strains accumulate the same level of cell-associated Cu (Fig. 7A and B). Lack of difference in cellular Cu content between the wild-type and *ΔtonB* mutant strains during Cu stress suggests that enterobactin is unlikely to be involved in importing Cu (Fig. 7A), unlike yersiniabactin which is reported to serve as a vehicle for Cu import in UPEC.^57^ The *Δfur* mutant overaccumulates Cu; however, the toxic effects of Cu are likely offset by the concurrent increase in Fe levels. Overaccumulation of Cu, without a concurrent increase in the level of Fe in the *copA* mutant explains increased sensitivity of this mutant to Cu that has been long known, and utilized as a control in our assays. Changes in cellular Fe content, taken in conjunction with increased expression of Fe-uptake genes during Cu stress reflects a mismatch between total and bioavailable levels of Fe and transcriptional repression activity of Fur collectively reflecting disruption of Fe homeostasis (Fig. 8). Our observations are aligned with the current model on mismetallation of noncognate proteins by Cu as a central mechanism of toxicity because maintaining a robust, bioavailable pool of cellular Fe is critical to ensure that Fe is incorporated into its cognate sites (Fig. 8).

## Conclusion

This report provides genome-level insights into the mechanisms involved in adaptation to Cu stress in *E. coli*. Here, we report the involvement of enterobactin pathway genes and several other genes in Cu-responsive phenotypes in *E. coli*. We provide evidence to support the roles of enterobactin-dependent Fe-uptake system genes in promoting *E. coli* survival during Cu stress. Collectively, our data supports a model in which both lack of enterobactin biosynthesis and failure to import enterobactin by TonB-dependent systems result in exacerbation of toxic effects of Cu (Fig. 8). Cellular metal content analysis indicates that Cu stress is associated with dysregulation of Fe homeostasis in *E. coli*. Our findings raise intriguing questions on the potential for direct interaction between Fur, enterobactin, and Cu, and the implication of such interactions on metal homeostasis. This report expands our knowledge on the link between homeostasis of transition metals in a bacterial cell and support observations at the host–pathogen interface, including in the urinary tract where concurrent overload of Cu and starvation for Fe is part of the innate immune response triggered during infection.^48,58^

## Supplementary Material

mfab052_Supplemental_Files

## Data Availability

The data underlying this article are available in the article and in its online supplementary material.
