# Supplementary material for: A genome-wide screen reveals the involvement of enterobactin-mediated iron acquisition in Escherichia coli survival during copper stress
Source: Metallomics. 2021 Aug 20;13(9):mfab052. doi: 10.1093/mtomcs/mfab052 (PMC8419524; doi:10.1093/mtomcs/mfab052)
Supplement: mfab052_Supplemental_Files [file mfab052_supplemental_files.zip › Supp Info.docx]

**Supplementary Table 1.** Bacterial strains and plasmids used in this study

| **Strain/Plasmid** | **Description^a^** | **Source** |  |
| --- | --- | --- | --- |
| ***E. coli*** |  |  |  |
| BW25113 | Wild-type strain | KEIO library (1) |  |
| BW25113 *ΔtonB* | *ΔtonB::npt* | KEIO library (1) |  |
| BW25113 *ΔexbB* | *ΔexbB::npt* | KEIO library (1) |  |
| BW25113 *ΔexbD* | *ΔexbD::npt* | KEIO library (1) |  |
| BW25113 *ΔfepB* | *ΔfepB::npt* | KEIO library (1) |  |
| BW25113 *ΔfepC* | *ΔfepC::npt* | KEIO library (1) |  |
| BW25113 *ΔfepD* | *ΔfepD::npt* | KEIO library (1) |  |
| BW25113 *ΔfepG* | *ΔfepG::npt* | KEIO library (1) |  |
| BW25113 *ΔentB* | *ΔentB::npt* | KEIO library (1) |  |
| BW25113 *ΔentE* | *ΔentE::npt* | KEIO library (1) |  |
| BW25113 *ΔentF* | *ΔentF::npt* | KEIO library (1) |  |
| BW25113 *ΔcopA* | *ΔcopA::npt* | KEIO library (1) |  |
| BW25113 *Δfur* | *Δfur::npt* | KEIO library (1) |  |
| BW25113/pGEN | Empty vector control | This study |  |
| BW25113 *ΔtonB*/pGEN | Empty vector control | This study |  |
| BW25113 *ΔtonB*/pGEN_*tonB* | *tonB* complementation | This study |  |
| **Uropathogenic *E. coli*** |  |  |  |
| CFT073 | Wild-type strain | (2) |  |
| CFT073 *ΔtonB* | *ΔtonB::npt* | (3) |  |
| CFT073 *ΔcopA* | *ΔcopA::npt* | (4) |  |
| CFT073/pGEN | Empty vector control | This study |  |
| CFT073 *ΔtonB*/pGEN | Empty vector control | This study |  |
| CFT073 *ΔtonB/*pGEN_*tonB* | *tonB* complementation | This study |  |
| CFT073 TN32-A2 | *c4289::Tn5,* copper resistant | Subash Lab^b^ |  |
| **Plasmids** |  |  |  |
| pGEN | Low-copy number vector | (5) |  |
| pGEN_*tonB* | *tonB* complementation | This study |  |

^a^*npt*, neomycin phosphortransferase; and *cat*, chloramphenicol acetyl transferase

^b^manuscript in preparation

**Supplementary Table 2.** Mutants lacking genes involved in Fe uptake/metabolism that were screened for Cu sensitivity

| **Gene** | **Function** | **Location** |
| --- | --- | --- |
| *fepA* | Ferric enterobactin outer membrane transporter | OM |
| *fecA* | Ferric enterobactin outer membrane transporter | OM |
| *cirA* | Ferric dihyroxybenzoylserine outer membrane transporter | OM |
| *fhuE* | Ferric coprogen/ferric rhodotorulic acid outer membrane transporter | OM |
| *fhuA* | Ferrichrome outer membrane transporter/phage receptor | OM |
| *fiu* | Iron catecholate outer membrane transporter | OM |
| *yncD* | Putative TonB-dependent outer membrane receptor | OM |
| *fhuD* | Iron(III) hydroxamate ABC transporter periplasmic binding protein | PP |
| *fepB* | Ferric enterobactin ABC transporter periplasmic binding protein | PP |
| *fecB* | Ferric citrate ABC transporter periplasmic binding protein | PP |
| *yncE* | PQQ-like domain-containing protein YncE | PP |
| *tonB* | Ton complex subunit TonB | PP/IM |
| *fhuC* | Iron(III) hydroxamate ABC transporter ATP binding subunit | IM |
| *exbB* | Ton complex subunit ExbB | IM |
| *fhuB* | Iron(III) hydroxamate ABC transporter membrane subunit | IM |
| *fepD* | Ferric enterobactin ABC transporter membrane subunit FebD | IM |
| *fepG* | Ferric enterobactin ABC transporter membrane subunit FepG | IM |
| *fepE* | Polysaccharide co-polymerase family protein FepE | IM |
| *fecD* | Ferric citrate ABC transporter membrane subunit FecD | IM |
| *fecE* | Ferric citrate ABC transporter ATP binding subunit | IM |
| *exbD* | Ton complex subunit ExbD | IM |
| *fecC* | Ferric citrate ABC transporter membrane subunit FecC | IM |
| *entE* | 2,3-dihydroxybenzoate-[aryl-carrier protein] ligase | IM/cytosol |
| *entB* | Enterobactin synthase component B | IM/cytosol |
| *fhuF* | Hydroxamate siderophore iron reductase | IM/cytosol |
| *fepC* | Ferric enterobactin ABC transporter membrane subunit FebD | IM/cytosol |
| *entF* | Apo-serine activating enzyme | IM/cytosol |
| *acnA* | Aconitate hydratase A | cytosol |
| *entA* | Enterobactin synthase component A | cytosol |
| *feoA* | Ferrous iron transport protein A | cytosol |
| *ftnA* | Ferritin iron-storage complex | cytosol |
| *fes* | Ferric enterobactin esterase | cytosol |
| *fur* | DNA-binding transcriptional dual regulator Fur | cytosol |
| *entC* | Isochorismate synthase EntC | cytosol |
| *ydiE* | PF10636 family protein YdiE | unknown |

^a^IM, inner membrane; PP, periplasm, and OM, outer membrane

**Supplementary Table 3.** Oligonucleotide primers used in this study

| **Primer ID^a^** | **Sequence** |
| --- | --- |
| **Purpose: Verification of KEIO library mutants** | |
| p469 *tonB* F | 5’GCGTTTTTCGAGGCTATCAG3’ |
| p470 *tonB* R | 5’AAGTATGTCGCGGTTGATCC3’ |
| p475 *fepA* F-Out | 5’TTAACGCCGTCACACCATAA3’ |
| p476 *fepA* R-In | 5’GCGATACAGACGGTTGGTTT3’ |
| p477 *fepB* F | 5’AGGGTTAATGTTCGCACCAG3’ |
| p478 *fepB* R | 5’GCGGTTTTGGTTTGTTGATT3’ |
| p485 *fepG* F | 5’GATTGCCTTTATTGGCCTGA3’ |
| p486 *fepG* R | 5’CAGATTTTCCGCAACGGTAT3’ |
| p487 *fes* F | 5’TAATTAATGTCCGCGCTTCC3’ |
| p488 *fes* R | 5’TCGACCAGCAAATGGTGATA3’ |
| p489 *entA* F | 5’GAGCATTTGATGTCGCTGAA3’ |
| p490 *entA* R | 5’CCATTGTGTTATCGCTGGTG3’ |
| p497 *entF* F-Out | 5’AATTTTGGGCGCAAAGTATG3’ |
| p498 *entF* R-In | 5’CGAATCAACCTCTCCGGTTA3’ |
| p499 *fepA* F-In | 5’AACGACCTTCACCTGGTACG3’ |
| p500 *fepA* R-Out | 5’TCCGGCTAAATGCTCTGTTT3’ |
| p501 *entF* F-In | 5’TGAAGGCAACTACGCTGATG3’ |
| p502 *entF* R-Out | 5’TTCCAGCAAGCTAACCGACT3’ |
| p503 *copA* F-Out | 5’CATTTTGTCCGCCGTTAAGT3’ |
| p504 *copA* R-In | 5’TATCAGGCCGATAACCAACC3’ |
| p505 *copA* F-In | 5’TGCAAAGTGAAGGACGTCAG3’ |
| p506 *copA* R-Out | 5’ACAAGAAAACCGACGACACC3’ |
| **Purpose: RT-qPCR** | |
| p554 *tonB* F | 5’GTGGCGGGTCTGCTCTATAC3’ |
| p555 *tonB* R | 5’TCGAGATCAGCAGGCGTAAC3’ |
| p556 *fepA* F | 5’AATCCGCAAAAACCCGGTTG3’ |
| p557 *fepA* R | 5’GGTTATTCCCACGCTGACCA3’ |
| p558 *fepB* F | 5’CTACCGCAACGCCCTTCTAT3’ |
| p559 *fepB* R | 5’GCCACGGCTGTCAGTAATCT3’ |
| p560 *fepG* F | 5’TGGTCACCGAATGGCGTTTA3’ |
| p561 *fepG* R | 5’TGAAAAATCGCGCCACTGAC3’ |
| p562 *fes* F | 5’CCAGTCGATGCAGCGAATTG3’ |
| p563 *fes* R | 5’TCGCGTTCGGTGGGAATAAA3’ |
| p564 *entA* F | 5’TTTGCGACCGAAGTGATGGA3’ |
| p565 *entA* R | 5’CGTAAAATTCCCGCCGCATT3’ |
| p566 *entF* F | 5’CAGAATTACCCTCCGCCTGG3’ |
| p567 *entF* R | 5’TATCTGCTTGCGCTAGTCCG3’ |
| p568 *copA* F | 5’TCACTCAGGCACGGGTAAAC3’ |
| p569 *copA* R | 5’TTCCACCGCCTGCACTAAAT3’ |
| p570 *gapA* F | 5’AACGTGATCCGGCTAACCTG3’ |
| p571 *gapA* R | 5’GCGGTGATGTGTTTACGAGC3’ |
| **Purpose: Verification of Downstream Gene Expression** | |
| p581 *entD* F | 5’CGGATCGCTGCTGTTTATGC3’ |
| p582 *entD* R | 5’TATCTGCCAGTGCACAGCAA3’ |
| p583 *fepC* F | 5’GCGAACAGTTAACCCTGGGA3’ |
| p584 *fepC* R | 5’GTTGTCCGCCAGAAAGGGTA3’ |
| p585 *ybdz* F | 5’TGGCATTCAGTAATCCCTTCGA3’ |
| p586 *ybdz* R | 5’TGGGTAAAATTCGTCGGTGTCA3’ |
| p587 *entF* F | 5’AATTTTGGGCGCAAAGTATG3’ |
| p588 *entF* R | 5’CGAATCAACCTCTCCGGTTA3’ |
| p589 *entH* F | 5’AACGCCATTTAACGCTCGAC3’ |
| p590 *entH* R | 5’GCCCCTGTTCATCGAAAACG3’ |
| p591 *fepE* F | 5’CCCGATTATCCTCTGGCGTC3’ |
| p592 *fepE* R | 5’CTGGCATTGTCATCAACCGC3’ |
| **Purpose: Verification of Genetic Complementation** | |
| p420 pGen F | 5’GGCACTTGCTCACGCTCTG3’ |
| p421 pGen R | 5’GTGGTCACGCTTTTCGTTGG3’ |

**Supplementary Table 4.** Cu-resistant mutants identified in the secondary screen

| **Gene** | **Function** | **Location^a^** |
| --- | --- | --- |
| *csgF* | Curli assembly component | OM |
| *tsx* | Nucleoside-specific channel-forming protein Tsx | OM |
| *lpp* | Murein lipoprotein | OM/PP |
| *zntA* | Zn^2+^/Cd^2+^/Pb^2+^ exporting P-type ATPase | IM |
| *ybgH* | Dipeptide:H+ symporter DtpD | IM |
| *ynaJ* | DUF2534 domain-containing protein Ynaj | IM |
| *nuoE* | NADH:quinone oxidoreductase subunit E | IM |
| *ydjN* | Cystine/sulfocysteine:cation symporter | IM |
| *yfdG* | Putative bactoprenol-linked glucose translocase | IM |
| *kdpF* | K+ transporting P-type atpase subunit KdpF | IM |
| *yqjF* | DoxX family protein | IM |
| *ybaK* | Cys-tRNApro and Cys-tRNAcys deacylase | cytosol |
| *rdgC* | Putative component of the Rsx system | cytosol |
| *sucC* | Succinyl-coA synthetase subunit β | cytosol |
| *pepT* | Peptidase T | cytosol |
| *rimJ* | Ribosomal-protein-S5-alanine N-acetyltransferase | cytosol |
| *cusR* | Transcriptional activator CusR | cytosol |
| *sucC* | Succinyl-coA synthetase subunit β | cytosol |
| *lon* | Lon protease | cytosol |
| *yehS* | DUF1456 domain-containing protein YehS | cytosol |
| *yciU* | DUF440 domain-containing protein YciU | cytosol |
| *ybiN* | 23S rRNA m6a1618 methyltransferase | cytosol |
| *rpiA* | Ribose-5-phosphate isomerase A | cytosol |
| *yddK* | Leucine-rich repeat domain-containing protein YddK | secreted |
| *ybfO* | RHS repeats-containing protein YbfO | Unknown |

^a^IM, inner membrane; PP, periplasm, and OM, outer membrane

**Supplementary Table 5.** Cu-sensitive mutants identified in the secondary screen

| **Gene** | **Function** | **Location** |
| --- | --- | --- |
| *ompC* | Outer membrane porin C | OM |
| *fepB* | Ferric enterobactin ABC transporter periplasmic binding protein | PP |
| *dcrB* | Periplasmic bacteriophage sensitivity protein DcrB | PP |
| *cueO* | Multicopper oxidase CueO | PP |
| *tonB* | Ton complex subunit TonB | PP/IM |
| *sdhD* | Succinate:quinone oxidoreductase, membrane protein SdhD | IM |
| *sdhA* | Succinate:quinone oxidoreductase, FAD binding protein | IM |
| *nuoF* | NADH:quinone oxidoreductase subunit F | IM |
| *sdhB* | Succinate:quinone oxidoreductase, Fe-S cluster binding protein | IM |
| *atpA* | ATP synthase F1 complex subunit α | IM |
| *atpC* | ATP synthase F1 complex subunit ε | IM |
| *atpH* | ATP synthase F1 complex subunit δ | IM |
| *copA* | Cu^+^ exporting P-type ATPase | IM |
| *atpD* | ATP synthase F1 complex subunit β | IM |
| *atpB* | ATP synthase F0 complex subunit a | IM |
| *fepD* | Ferric enterobactin ABC transporter membrane subunit FepD | IM |
| *exbD* | Ton complex subunit ExbD | IM |
| *exbB* | Ton complex subunit ExbB | IM |
| *tolR* | Tol-Pal system protein TolR | IM |
| *fepG* | Ferric enterobactin ABC transporter membrane subunit FepG | IM |
| *fepD* | Ferric enterobactin ABC transporter membrane subunit FepD | IM |
| *exbB* | Ton complex subunit ExbB | IM |
| *tolR* | Tol-Pal system protein TolR | IM |
| *yebA* | Peptidoglycan DD-endopeptidase MepM | IM |
| *gltA* | Citrate synthase | cytosol |
| *ubiF* | 2-octaprenyl-3-methyl-6-methoxy-1,4-benzoquinol hydroxylase | cytosol |
| *gor* | Glutathione reductase | cytosol |
| *ubiX* | Flavin prenyltransferase | cytosol |
| *yfjG* | Ribosome association toxin RatA | cytosol |
| *astE* | Succinylglutamate desuccinylase | cytosol |
| *secB* | SecB chaperone | cytosol |
| *purA* | Adenylosuccinate synthetase | cytosol |
| *cpxR* | DNA-binding transcriptional dual regulator CpxR | cytosol |
| *ompR* | Transcriptional dual regulator | cytosol |
| *pdxH* | Pyridoxine 5'-phosphate oxidase / pyridoxamine 5'-phosphate oxidase | cytosol |
| *cyaA* | Adenylate cyclase | cytosol |
| *iscS* | Cysteine desulfurase | cytosol |
| *rpsF* | 30S ribosomal subunit protein S6 | cytosol |
| *ubiH* | 2-octaprenyl-6-methoxyphenol 4-hydroxylase | cytosol |
| *rbfA* | 30S ribosome binding factor | cytosol |
| *rpmJ* | 50S ribosomal subunit protein L36 | cytosol |
| *guaA* | GMP synthetase | cytosol |
| *lipB* | lipoyl(octanoyl) transferase | cytosol |
| *pnp* | Polynucleotide phosphorylase | cytosol |
| *rsgA* | Ribosome small subunit-dependent GTPase A | cytosol |
| *rimM* | Ribosome maturation factor RimM | cytosol |

^a^IM, inner membrane; PP, periplasm, and OM, outer membrane

**Supplementary Table 6.** Expression of downstream genes in KEIO mutants

| **KEIO Mutant** | **Downstream Gene** | **Expression (+/-)^a^** |
| --- | --- | --- |
| *ΔfepG* | *fepC* | + |
| *Δfes* | *ybdZ* | + |
| *Δfes* | *entF* | + |
| *ΔentA* | *entH* | + |
| *ΔentF* | *fepE* | + |

^a^Based on RT-PCR

**Supplementary Figure Legends**

**Figure S1. Operons involved in enterobactin production and uptake in *E. coli*.** Schematic of gene organization as operons including the location of promoters and Fur binding sites was generated in Biorender, based on publicly available information from EcoCyc.org.

**Figure S2. Copper toxicity in spent medium from the wild-type strain.** Wild-type (BW25113), *ΔtonB,* and *ΔentF* mutant strains were cultured in spent LB harvested from stationary phase cultures of wild-type strain, that was supplemented with 0 (solid bars) or 3 mM CuSO_4_ (hatched bars). Viable counts were determined after 24 hours from three biological replicates. Bars indicate median and error bars indicate interquartile range. **P*<0.05; and ***P*<0.01 by Kruskal-Wallis test with Dunn’s post-test.

**Figure S3. Concentration of select transition metals in *E. coli*.** Wild-type (BW25113), *ΔtonB, Δfur,* and *ΔcopA* mutant strains were cultured in LB or LB supplemented with 3 mM CuSO_4_. Cell pellets were digested and analyzed by ICP-MS or -OES to quantify Zn (**A** & **B**), and Mn (**C** & **D**). Mean+SEM from three biological replicates is presented here.

**REFERENCES**

1. Baba T, Ara T, Hasegawa M, Takai Y, Okumura Y, Baba M, Datsenko KA, Tomita M, Wanner BL, Mori H. 2006. Construction of Escherichia coli K-12 in-frame, single-gene knockout mutants: the Keio collection. Mol Syst Biol 2:2006 0008.

2. Mobley HL, Green DM, Trifillis AL, Johnson DE, Chippendale GR, Lockatell CV, Jones BD, Warren JW. 1990. Pyelonephritogenic Escherichia coli and killing of cultured human renal proximal tubular epithelial cells: role of hemolysin in some strains. Infect Immun 58:1281-9.

3. Yep A, McQuade T, Kirchhoff P, Larsen M, Mobley HL. 2014. Inhibitors of TonB function identified by a high-throughput screen for inhibitors of iron acquisition in uropathogenic Escherichia coli CFT073. MBio 5:e01089-13.

4. Saenkham P, Ritter M, Donati GL, Subashchandrabose S. 2020. Copper primes adaptation of uropathogenic Escherichia coli to superoxide stress by activating superoxide dismutases. PLoS Pathog 16:e1008856.

5. Lane MC, Alteri CJ, Smith SN, Mobley HL. 2007. Expression of flagella is coincident with uropathogenic Escherichia coli ascension to the upper urinary tract. Proc Natl Acad Sci U S A 104:16669-74.
