# Supplementary figures and images for: A genome-wide screen reveals the involvement of enterobactin-mediated iron acquisition in Escherichia coli survival during copper stress
Source: Metallomics. 2021 Aug 20;13(9):mfab052. doi: 10.1093/mtomcs/mfab052 (PMC8419524; doi:10.1093/mtomcs/mfab052)

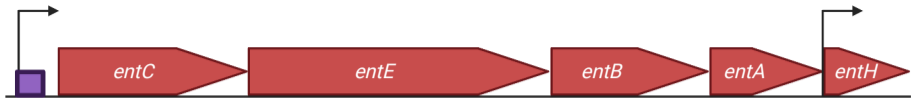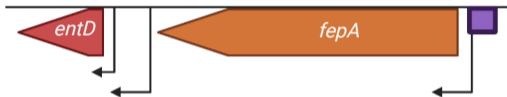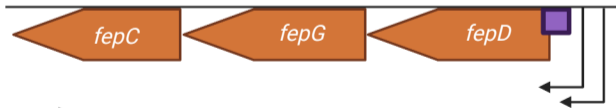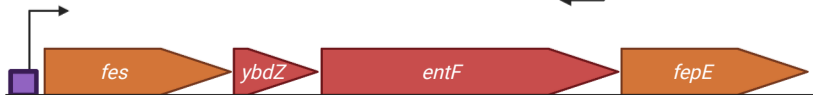

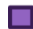 Fur box

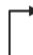 Promoter

Supplement: mfab052_Supplemental_Files [file mfab052_supplemental_files.zip › Fig. S1.pdf]

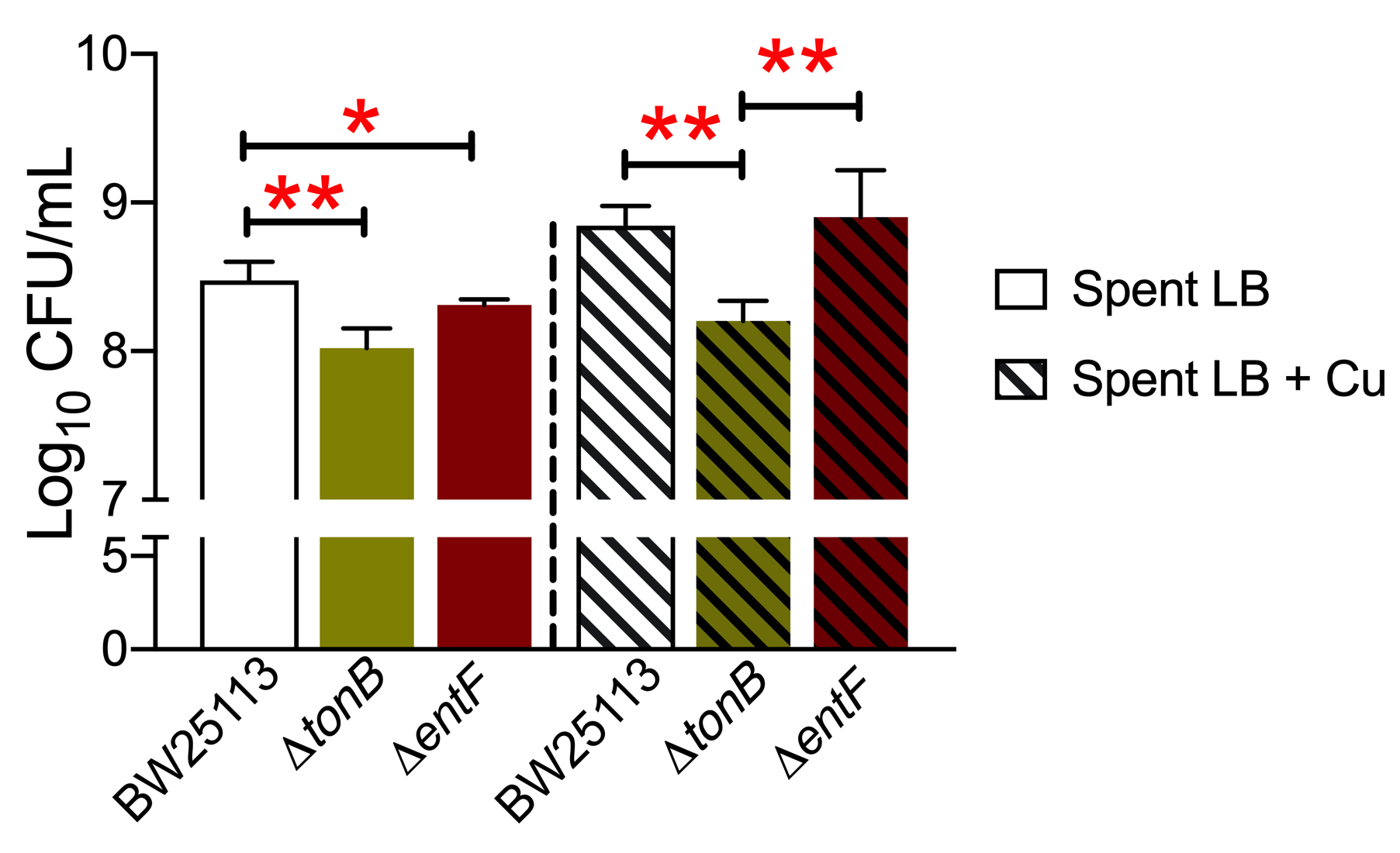

Supplement: mfab052_Supplemental_Files [file mfab052_supplemental_files.zip › Fig. S2.tiff]

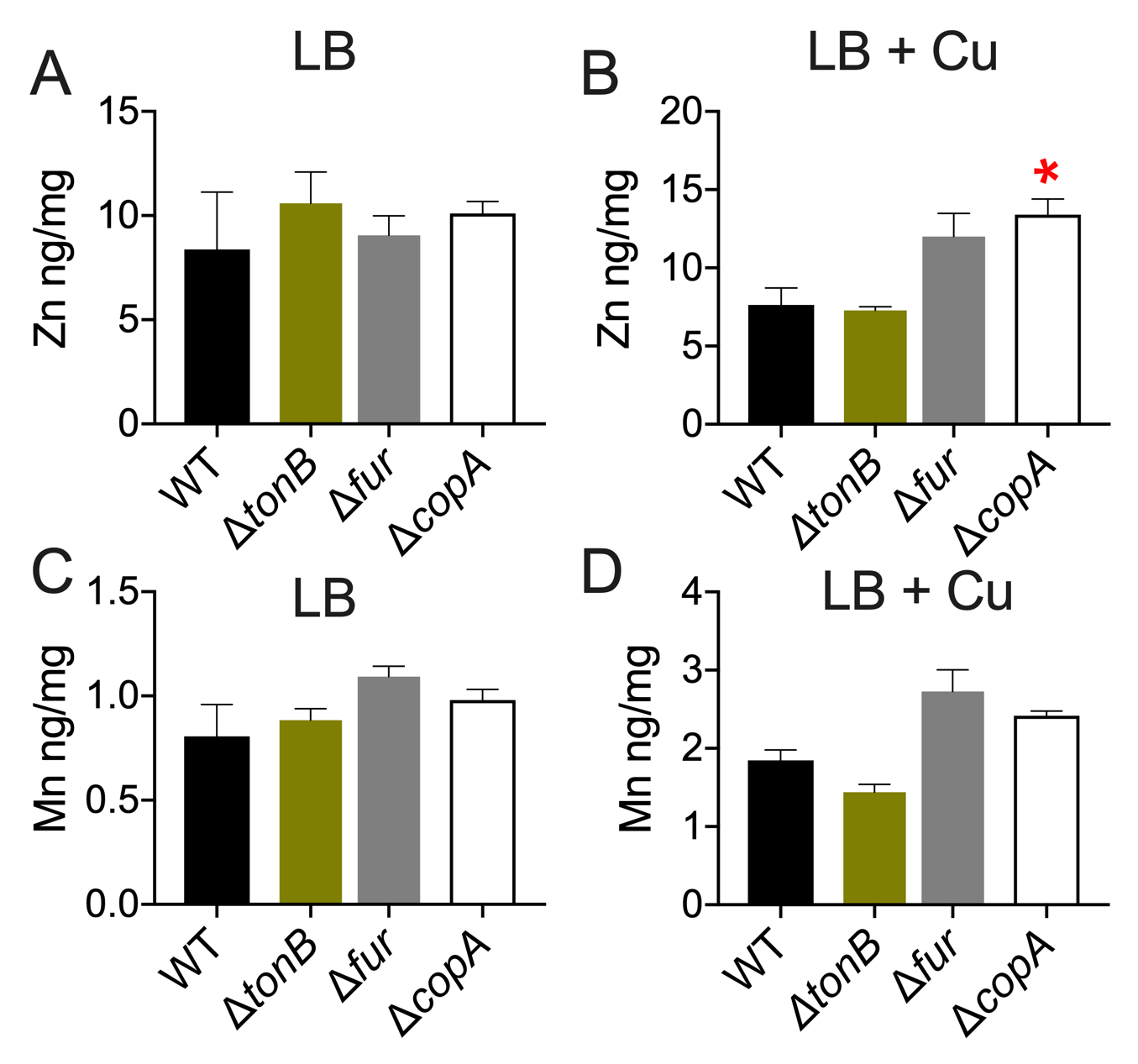

Supplement: mfab052_Supplemental_Files [file mfab052_supplemental_files.zip › Fig. S3.tiff]
